# Coenzyme Q10 combined with trimetazidine in the prevention of contrast-induced nephropathy in patients with coronary heart disease complicated with renal dysfunction undergoing elective cardiac catheterization: a randomized control study and in vivo study

**DOI:** 10.1186/s40001-018-0320-2

**Published:** 2018-05-18

**Authors:** Fei Chen, Fan Liu, Jingchao Lu, Xiuchun Yang, Bing Xiao, Yaqiong Jin, Jie Zhang

**Affiliations:** Department of Cardiology, The Second Hospital of Hebei Medical University, No. 215 Hepingxi Road, Xinhua District, Shijiazhuang, 050005 Hebei China

**Keywords:** Percutaneous coronary intervention, Contrast-induced nephropathy, Coenzyme Q10, Trimetazidine, Prevention, Animal model

## Abstract

**Background:**

Contrast-induced nephropathy (CIN) is one of the common hospital-acquired acute renal failures. The purpose of this study was to investigate whether Coenzyme Q10 (CoQ10) and trimetazidine (TMZ) can prevent the occurrence of CIN after elective cardiac catheterization in patients with coronary artery disease complicated with renal dysfunction.

**Methods:**

Consecutive coronary artery disease patients with renal insufficiency scheduled for coronary angiography were enrolled in randomized, paralleled, double-blind, controlled trial. The development of CIN was occurrence at the 48 or 72 h after the procedure. The changes of serum creatinine (SCr), eGFR, and Cys-C within 72 h after the procedure were measured and compared. In vivo contrast medium (CM)-induced acute kidney injury (AKI) animal model was established, and CoQ10 plus TMZ was orally administrated to evaluate its renal protective effect.

**Results:**

150 patients with renal insufficiency were enrolled finally. CIN occurred in 21 (14.00%) of the 150 patients. 72 h after the procedure, the incidence of CIN was significantly lower in CoQ10 plus TMZ group compared with control group (6.67 vs. 21.3%, *p* = 0.01). No cardiac death occurred in this study. No side effects were observed after administration of CoQ10 and TMZ. In vivo test demonstrated that CoQ10 and TMZ could significantly reduce the concentration of blood urea nitrogen (BUN) and SCR induced by CM *i.v.* injection, as well as tubular pathological injuries. Meanwhile, CoQ10 and TMZ could significantly reduce the oxidation stress in kidneys from CM-AKI animals.

**Conclusion:**

CoQ10 plus TMZ could decrease the incidence of CIN in patients with renal insufficiency undergoing elective cardiac catheterization, and their effect may be due to its strong anti-oxidation effect.

## Background

Percutaneous coronary intervention (PCI) is one of the most effective therapies for coronary heart disease. With the improvement of interventional technique of coronary artery in recent years, the number of percutaneous coronary intervention was increasing [1–3]. Treating complicated coronary artery disease, especially chronic occlusion disease (CTO), is not a difficult problem presently [4]. Contrast agents are often used in coronary interventions, especially a large number of contrast agents are used in complex coronary lesions and CTO. However, using of contrast agents in clinical coronary angiography and PCI treatment could cause acute renal impairment (AKI), especially for patients with pre-existing renal failure [5, 6].

Contrast-induced nephropathy (CIN) is one of the commonly acquired acute renal failures in hospital, which could cause higher mortality, higher treatment costs, and prolonged hospitalization [7, 8]. The clinical and basic research of CIN has become the hot spot currently. Hydration and maintenance of circulating volume are still the main measurement to prevent contrast nephropathy [9]. Clinical studies also showed that several drugs can prevent contrast nephropathy, including antioxidants (*N*-acetylcysteine, statins), diuretic (furosemide), and vasodilators (such as calcium antagonists, dopamine, fenoldopam) [10, 11]. However, the therapeutic effect of these drugs is uncertain, and still needs to be verified in larger clinical trials.

Studies have shown that reactive oxidative stress and oxygen species (ROS) play an important role in the apoptosis of renal tubular epithelial cells induced by contrast media [12]. As a strong antioxidant, Coenzyme Q10 (CoQ10) could alleviate oxidative stress-induced damage in cell and mitochondrial membrane. Several researches also demonstrate CoQ10 has cardiac protective effect for patients with coronary heart disease [13]. Trimetazidine (TMZ) hydrochloride could maintain the energy metabolism of cells under hypoxia or ischemia condition. It could increase coronary blood flow reserve and reduce the frequency of angina pectoris significantly in patients with coronary heart disease [14].

Based on their pharmacological effect of CoQ10 and TMZ, we hypothesize that CoQ10 and TMZ may reduce the risk of CIN. The purpose of this study was to investigate whether combination of CoQ10 and TMZ can prevent the occurrence of CIN after elective cardiac catheterization in patients with coronary artery disease complicated with renal insufficiency. After clinical trial, we established a contrast-induced acute kidney injury (CI-AKI) in rats, and CoQ10 and TMZ was administrated orally to assess their renal protective effect.

## Methods

The study protocol was approved by the ethics committee of the second Hospital of Hebei Medical University. Informed consent was obtained on admission. The study was conducted in accordance with the Declaration of Helsinki. The study was registered in Chinese Clinical Trial Registry with the serial number, ChiCTR-INR-17010449.

### Study population

This study was a randomized, paralleled, double-blind, controlled trial. From Jan 2016 to Aug 2017, 180 consecutive coronary artery disease (CAD) patients with renal insufficiency scheduled for coronary angiography (CAG) were enrolled in cardiology department, the second hospital of Hebei Medical University. The inclusion criteria were age ≥ 18 years, males or females, patients with coronary heart disease who would subsequently undergo elective cardiac catheterization and estimated glomerular filtration rate (eGFR) ≤ 60 mL/min/1.73 m^2^. Exclusion criteria: current Coenzyme Q10 and trimetazidine (TMZ) treatment; high-risk features warranting emergency coronary angiography (within 4 h); renal failure requiring dialysis; contrast agent exposure within the previous 7 days; left ventricular ejection fraction (LVEF) < 30%; administration of *N*-acetylcysteine within 48 h of the procedure and refusal of consent. The renal insufficiency was defined as an estimated glomerular filtration rate (eGFR) ≤ 60 mL/min/1.73 m^2^. The eGFR was calculated by serum creatinine (SCr) levels and the modification of diet in renal disease (MDRD) equation:

$${\text{eGFR }}\left( {{\text{mL}}/{ \hbox{min} }/1.73\,{\text{m}}^{2} } \right) = 186 \times {\text{SCr}}^{ - 1.154} \times {\text{age}}^{ - 0.203} \left( { \times 0.742\,{\text{for}}\,{\text{female}}\,{\text{subjects}}} \right).$$

### Study protocol

**Fig. 1:**
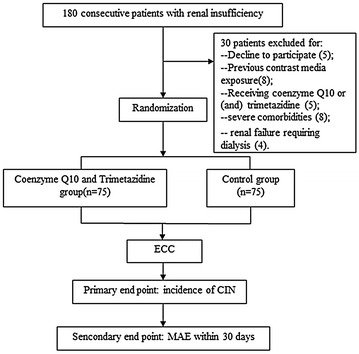
Flow diagram of the study. *ECC* elective cardiac catheterization, *CIN* contrast-induced nephropathy, *MAE* major adverse event

Recruited patients were randomly assigned to CoQ10 [Eisai (china) Pharmaceutical Co. Ltd, Suzhou, China] plus TMZ [Servier (Tianjin) Pharmaceutical Co. Ltd, Tianjin, China] group or control group by a 1:1 ratio using computer-generated random numbers (Fig. 1). In CoQ10 plus TMZ group, both of CoQ10 and TMZ were administered 20 mg three times daily from 2 days before to 3 days after procedure. Control group were given same packaged placebo as the same method.

Coronary angiography and percutaneous coronary intervention (PCI) were performed by radial or femoral approach. The procedure was performed by the same medical team in both groups. The contrast agent (Ultravist 370, iodine 370 mg/mL, Schering Pharmaceutical Ltd., China) was used for all patients. All patients were treated with hydration as below: an intravenous infusion of 0.9% saline at a rate of 1 or 0.5 mL/kg/h (patients with LVEF < 40%) 4 h before and 20 h after elective coronary procedure.

During the PCI, An intravenous bolus of unfractionated heparin (70–100 U/kg) or bivalirudin (0.75 mg/kg injected intravenously as a load dose before PCI, continuous infusion of 1.75 mg/kg/h for no more than 4 h) was given for anticoagulation. The platelet glycoprotein IIb/IIIa inhibitors (GPI) were administered according to condition of coronary lesions. Moreover, aspirin (100 mg/day) and clopidogrel (75 mg/day) were routinely administrated to prevent platelet aggregation before and after the procedure. If the patient received PCI, dual antiplatelet therapy continued for at least 12 months unless severe bleeding complications occurred. Before randomization, and at 48, 72 h after contrast agent exposure, blood samples were collected to measure the levels of SCr with the Hitachi 7600 automated biochemistry analyzer (Hitachi, Ltd., Tokyo, Japan) and Cystatin-C (Cys-C) with the Mindray BS-800 automated biochemistry analyzer (Shenzhen Mindray Bio-Medical Electronics Co., Ltd, Shenzhen, China).

### Study endpoints

The primary endpoint was development of CIN, 48 or 72 h after the procedure. CIN defined as 25% relative increase in SCr from baseline or absolute increase of 44 μmol/L (0.5 mg/dL) after exposure to contrast medium. The secondary endpoints were (1) the changes of SCr, eGFR, and Cys-C within 72 h after the procedure; (2) major adverse events occurring within 30 days after contrast medium exposure were recorded, including all-cause death, myocardial infarction, renal failure requiring dialysis, stroke, upper gastrointestinal bleeding, and worsening heart failure which was defined as a deteriorated NYHA (New York Heart Association) functional class. All patients had a follow-up in out-patient clinic or contacted by telephone at 30 days.

### Induction of CI-AKI and experimental treatment

Male Sprague–Dawley rats (180–200 g) were purchased from Hei Bei medical university (Shijiazhuang, Hebei, China). The rats were kept in individual cages under controlled conditions at 20–24 °C on a 12:12-h light/dark cycle and had free access to tap water and a standard laboratory diet. All experimental protocols were approved by the Committee on the Ethics of Animal Experiments of the School of Medicine, Hebei medical University (HM-20170111) and were in compliance with the Guide for the Care and Use of Laboratory Animals by the National Academy Press.

CI-AKI was induced by using a previously reported method [15]. In brief, rats were deprived of water for 72 h and then given 10 mg/kg furosemide (Harvest Pharmaceutical Co., Shanghai, China) by intramuscular injection. After 20 min, a non-ionic, low-osmolar contrast media (CM, Omnipaque, 350 mg I/mL; GE Healthcare, Shanghai, China) was administered by intravenous injection (10 mL/kg) via the tail vein over the course of 5 min.

Forty-five rats were randomly allocated to the following five groups (*N* = 8 per group): (1) control group (dehydration + furosemide, without CM administration); (2) CI-AKI group; (3) CI-AKI + CoQ10 and TMZ group (10 mg/kg for both drugs); (4) CI-AKI + CoQ10 and TMZ group (20 mg/kg for both drugs). Baseline blood samples were collected from the jugular vein under light ether anesthesia at the end of acclimatization period, and the serum was separated from the whole blood. CoQ10 and TMZ were administered by oral gavage once daily for three consecutive days prior to CM injection and once at 4 h after CM injection. The rats were allowed to recover for 24 h after the CM injection and then sacrificed by light ethyl ether anesthesia. The final blood samples were collected through the jugular vein. After harvesting the kidneys, the left kidney was stored at − 80 °C for biochemical analysis, and the right kidney was fixed in 10% formalin for histopathological evaluation.

### Biochemical test for renal Function

Blood urea nitrogen (BUN) and serum creatinine (SCr) concentration was determined using an automatic biochemical analyzer (Hitachi 7600, Japan) at the central clinical laboratory of second hospital of Hebei Medical University.

### Histopathological analysis of kidney tissues

Kidney tissue was fixed in 10% neutral buffered formalin, embedded in paraffin, cut into 3 μm sections, and stained with hematoxylin and eosin (H&E). Histopathological analysis was performed in a blinded manner using a light microscope (Olympus, Japan). Ten high-magnification (× 200) fields of the cortex and outer stripe of the outer medulla were randomly selected for semiquantitative analysis.

The renal lesions were graded according to following criterions: Tubular necrosis and proteinaceous casts were graded as follows: 0 = no damage, 1 = mild (unicellular, patchy isolated damage), 2 = moderate (< 25% damage), 3 = severe (25–50% damage), or 4 = very severe (> 50% damage).

### Measurement of oxidative stress markers in vivo and in vitro

Kidneys were homogenized in PBS using a Tissuelyser-38 (Jingxin industrial development co. Ltd, Shanghai, China) and then centrifuged at 3000*g* for 10 min. The pellet was discarded and protein content was measured in supernatant using the bicinchoninic acid (BCA) assay (Applygen Technologies Inc. Beijing, China). Supernatant aliquots were used to determine the oxidative markers, including nitric oxide (NO), glutathione (GSH), lipid peroxidation (LPO), activity of superoxide dismutase (SOD), and catalase (CAT). The test procedure was followed as specifications of each commercial kits (Nanjing Jiancheng biotechnology, Nanjing, China).

### Statistical analysis

According to previous research, it is speculated that the incidence of CIN after the procedure was 20% in control group, and we hypothesized the CoQ10 plus TMZ group could reduce the incidence of CIN to 5%. In view of the above, at least 73 patients each group were required for the power of the test set at 0.8 and statistical level (2-sided) at 0.05. The continuous variables were expressed as means with standard deviation for normally distributed variables while as median with interquartile range for non-normally distributed variables. The categorical variables were presented as percentage. Continuous variables were compared using the Student t test for normally distributed value and the Mann–Whitney *U* test for non-normally distributed. Proportions were compared using the Chi-square test, and if the expected frequency was < 5, the Fisher exact test would be applied. Analysis of variance (ANOVA) was used to compare the difference of SCr, eGFR, and Cys-C levels before and after the procedure in each group. Multivariate logistic regression analysis was used to explore the possible factors associated with the incidence of CIN. For the in vivo animal study, all measurement data were presented as mean ± standard error (SEM), Comparison of the same parameters among all groups was done using one-way analysis of variance (ANOVA), and the difference between pairs of means was tested post hoc with Tukey’s test. *p* < 0.05 was considered statistically significant. All calculations were analyzed with the SPSS statistical software (version 17.0, SPSS Inc., Chicago, IL).

## Results

According to the inclusion criteria, a total of 180 patients were enrolled initially. 30 patients were excluded, who declined to participate (5), whose previous contrast media exposure was within 7 days (8), who received coenzyme Q10 or (and) TMZ within 1 week (5), had severe comorbidities (8), and had renal failure requiring dialysis (4). Finally, a total of 150 patients with renal insufficiency were enrolled and were randomly divided into the CoQ10 plus TMZ group (*n* = 75) and control group (*n* = 75) (Fig. 1).

### Baseline characteristics

Baseline characteristics of the patients were similar in the two groups (Table 1). There were no significant differences between the two groups about age, gender, body mass index (BMI), hypertension, diabetes mellitus, hyperlipidemia, smoking, LVEF, laboratory results, medications, used of bivalirudin, and GPI. There were 39 patients (52.00%) in the CoQ10 plus TMZ group and 36 patients (48.00%) in the control group which underwent PCI (*p* = 0.430). The volume of the contrast medium used during the coronary procedure was 136.40 ± 68.57 mL in the CoQ10 plus TMZ group and 130.13 ± 63.79 mL in the control group (*p* = 0.563).

**Table 1:** Baseline clinical characteristics of the patients

Variables	CoQ10 + TMZ group (*n* = 75)	Control group (*n* = 75)	*p* value
Age (years)	61.75 ± 9.15	63.52 ± 7.88	0.205
Female, *n* (%)	34 (45.33)	33 (44.00)	0.870
BMI (kg/m^2^)	26.30 ± 4.06	25.43 ± 2.85	0.131
Hypertension, *n* (%)	32 (42.67)	35 (46.67)	0.622
Diabetes mellitus, *n* (%)	31 (41.33)	34 (45.33)	0.621
Hyperlipidemia, *n* (%)	29 (38.67)	26 (34.67)	0.611
Smoking, *n* (%)	39 (52.00)	41 (54.67)	0.743
LVEF (%)	56.18 ± 6.53	54.62 ± 5.68	0.121
AMI, *n* (%)	13 (17.33)	10 (13.33)	0.479
Previous myocardial infarction *n* (%)	11 (14.67)	8 (20.67)	0.461
Laboratory results			
Hemoglobin (g/L)	125.01 ± 9.28	126.25 ± 9.72	0.426
Glycosylated hemoglobin (%)	6.08 ± 0.88	6.26 ± 1.00	0.229
Low-density lipoprotein cholesterol (mg/dL)	118.79 ± 18.45	117.75 ± 21.39	0.750
Medications, *n* (%)			
β-blocker	52 (69.33)	49 (65.33)	0.601
ACEI/ARB	42 (56.00)	38 (50.67)	0.513
Statins	67 (89.33)	71 (94.67)	0.229
Diuretics	6 (8.00)	8 (10.67)	0.575
Nitrate	40 (53.33)	46 (61.33)	0.322
Calcium-channel blocker	32 (42.67)	38 (50.67)	0.326
Proton pump inhibitors	20 (26.67)	16 (21.33)	0.444
PCI, *n* (%)	39 (52.00)	36 (48.00)	0.624
Use of bivalirudin, *n* (%)	26 (34.67)	21 (28.00)	0.379
Use of GPI, *n* (%)	15 (20.00)	16 (21.33)	0.840
Volume of CM (mL)	136.40 ± 68.57	130.13 ± 63.79	0.563
CM ≥ 160 mL, *n* (%)	35 (46.67)	30 (40.00)	0.410
Hydration volume (mL)	1754.40 ± 358.41	1716.32 ± 238.30	0.445

*BMI* Body mass index, *AMI* acute myocardial infarction, *LVEF* left ventricular ejection fraction, *ACEI* angiotensin-converting enzyme inhibitors, *ARB* angiotensin receptor blockers, *PCI* percutaneous coronary intervention, *GPI* platelet glycoprotein IIb/IIIa inhibitors, *CM* contrast medium

### The changes of renal function parameters and the incidence of CIN

The change levels of renal function parameters (SCr, eGFR, and Cys-C) were compared between the two groups (Table 2). There were no significant differences of SCr (118.18 ± 17.27 vs. 119.38 ± 14.81 μmol/L, *p* = 0.647), eGFR (51.28 ± 5.03 vs. 50.36 ± 4.15 mL/min/1.73 m^2^, *p* = 0.223), and Cys-C (1.51 ± 0.26 vs. 1.53 ± 0.20 mg/L, *p* = 0.503) on baseline between the CoQ10 plus TMZ group and control group. The levels of SCr and Cys-C increased at 48 h and declined at 72 h after procedure in both groups. Significant rise was observed in SCr and Cys-C levels at 48 and 72 h (*p* < 0.05) in control group compared with the baseline; Simultaneously, CoQ10 plus TMZ group only increased significantly in 48 h (*p* < 0.05). CoQ10 plus TMZ group tended to have a lower SCr and Cys-C levels than control group at 48 and 72 h after the procedure (all *p *< 0.05). The eGFR decreased significantly after the procedure in both groups, with the lowest value occurring at 48 h and then beginning to recover at 72 h. Compared with the control group, CoQ10 plus TMZ group tended to have a higher eGFR levels at 48 and 72 h after the procedure (46.30 ± 5.69 vs. 43.14 ± 5.81 mL/min/1.73 m^2^, *p* = 0.001; 50.70 ± 5.57 vs. 48.50 ± 5.24 mL/min/1.73 m^2^, *p* = 0.014).

**Table 2:** Changes of SCr, eGFR, and Cystatin-C, incidence of CIN

Variables	CoQ10 + TMZ group (*n* = 75)	Control group (*n* = 75)	*p* value
SCr (μmol/L)			
Baseline	118.18 ± 17.27	119.38 ± 14.81	0.647
48 h after exposure	129.86 ± 21.75*	139.52 ± 20.21*	0.005
72 h after exposure	118.97 ± 16.82	125.06 ± 15.11*	0.021
eGFR (mL/min/1.73 m^2^)			
Baseline	51.28 ± 5.03	50.36 ± 4.15	0.223
48 h after exposure	46.30 ± 5.69*	43.14 ± 5.81*	0.001
72 h after exposure	50.70 ± 5.57	48.50 ± 5.24*	0.014
Cystatin-C (mg/L)			
Baseline	1.51 ± 0.26	1.53 ± 0.20	0.503
48 h after exposure	1.95 ± 0.43*	2.13 ± 0.35*	0.006
72 h after exposure	1.55 ± 0.27	1.64 ± 0.21*	0.016
Incidence of CIN, *n* (%)	5 (6.67)	16 (21.3)	0.010

*SCr* serum creatinine, *eGFR* estimated glomerular filtration rate, *CIN* contrast-induced nephropathy

* *p* < 0.05 compared with baseline

Overall, CIN occurred in 21 (14.00%) of the 150 patients. Within 72 h after the procedure, the incidence of CIN was significantly lower in CoQ10 plus TMZ group compared with control group (6.67 vs. 21.3%, *p* = 0.01).

### Predictors of CIN by multiple logistic regression analysis

Multiple logistic regression analysis identified administration of CoQ10 plus TMZ as strong predictor of decreased risk of CIN (OR 0.252, 95% CI 0.082–0.774, *p* = 0.016). In addition, the results showed that the volume of contrast medium ≥ 160 mL (OR 5.570 95% CI 1.693–18.327, *p* = 0.005) and eGFR ≤ 45 mL/min/1.73 m^2^ (OR 3.010, 95% CI 1.058–8.556, *p* = 0.039) were independent predictors of CIN after coronary procedure (Table 3).

**Table 3:** Predictors of CIN by multivariate logistic analysis

Variables	OR	95% CI	*p* value
CoQ10 + TMZ	0.252	0.082–0.774	0.016
Sex	0.761	0.221–2.623	0.665
Age **> **65	2.119	0.696–6.457	0.186
LVEF (%)	1.075	0.976–1.185	0.143
BMI	1.046	0.856–1.278	0.661
Diabetes	1.111	0.369–3.348	0.851
Hemoglobin	0.967	0.906–1.032	0.308
Contrast volume ≥ 160 mL	5.570	1.693–18.327	0.005
eGFR ≤ 45 mL/min/1.73 m^2^	3.010	1.058–8.556	0.039

*CIN* Contrast-induced nephropathy, *eGFR* estimated glomerular filtration rate

### Clinical endpoints

One-month clinical follow-up was obtained in 148 of 150 patients (98.7%). The incidence of major adverse events has no significant difference between the two groups (6.76% vs. 8.1%, *p *= 0.754). There were 2 cases of worsening heart failure, 1 case stroke, 1 case upper gastrointestinal bleeding, and 1 case developed clinical renal failure occurred in the CoQ10 plus TMZ group after contrast exposure. In control group, there were 3 cases of worsening heart failure, 1 case myocardial infarctions, and 2 cases needed hemodialysis. None of the patients incurred cardiac death in this study. After administration of CoQ10 and TMZ, no side effects were observed.

### CoQ10 and TMZ reduces renal dysfunction in rat with CM-induced renal injury

To investigate the effects of CoQ10 and TMZ on cisplatin-induced renal dysfunction, the serum levels of BUN and creatinine in rat were measured 24 h after CM injection. CM challenge elevated serum BUN and creatinine levels to 57.1 ± 5.67 and 0.63 ± 0.06 mg/dL, respectively, which was significantly higher than the normal control (26.3 ± 0.82 and 0.38 ± 0.07 mg/dL, respectively; Table 4). The CoQ10 and TMZ treatment significantly decreased BUN and creatinine levels compared to CM model control in a dose-dependent manner (Table 4).

**Table 4:** Blood urea nitrogen and serum creatinine at the post-treatment with the CoQ10 and TMZ in CM-induced AKI model

Groups	sham	CM	CM + CoQ10 and TMZ (10 mg/kg)	CM + CoQ10 and TMZ (20 mg/kg)
*N*	8	8	8	8
BUN (mg/dL)	26.3 ± 0.82	57.1 ± 5.67^#^	44.3 ± 4.38*	33.1 ± 3.21**
Scr (mg/dL)	0.38 ± 0.07	0.63 ± 0.06^#^	0.51 ± 0.09*	0.43 ± 0.05*

^#^ *p* < 0.05, compared with sham control; * *p* < 0.05, ** *p* < 0.01, compared with CM model group

### CoQ10 and TMZ reduced the oxidation in the CM-induced kidney tissues

As shown in Table 5, CM challenge caused significantly higher oxidative stress in the kidney tissues, which led to lower level of GSH and higher production of LPO, as well as decrease in antioxidant enzyme activities, such as SOD and Catalase. In the current study, CoQ10 and TMZ administration significantly ameliorated the oxidative stress in the CM-challenged kidney tissues by increasing the GSH level, SOD and catalase activities, while decreasing the LPO contents.

**Table 5:** Effect of CoQ10 and TMZ on oxidative biomarkers in CM-induced acute kidney injury

Groups	Sham control	CM	CM + CoQ10 and TMZ (10 mg/kg)	CM + CoQ10 and TMZ (20 mg/kg)
N	8	8	8	8
GSH (mmol/g tissue)	9.18 ± 1.21	2.69 ± 0.14^#^	5.47 ± 0.10*	6.42 ± 0.39*
MDA (nmol/g tissue)	21.5 ± 2.12	61.1 ± 2.34	45.6 ± 2.47*	30.9 ± 3.41**
Catalase (*U*/mg protien)	10.50 ± 0.68	4.42 ± 0.37^#^	6.69 ± 0.52	8.27 ± 0.30**
SOD (*U*/mg protein)	14.42 ± 0.48	7.36 ± 1.53^#^	10.49 ± 0.54*	12.55 ± 0.76*

^#^*p* < 0.05, compared with sham control; * *p* < 0.05, ** *p* < 0.01, compared with CM model group

### CoQ10 and TMZ ameliorate the renal tubular impairment on pathological analysis

**Fig. 2:**
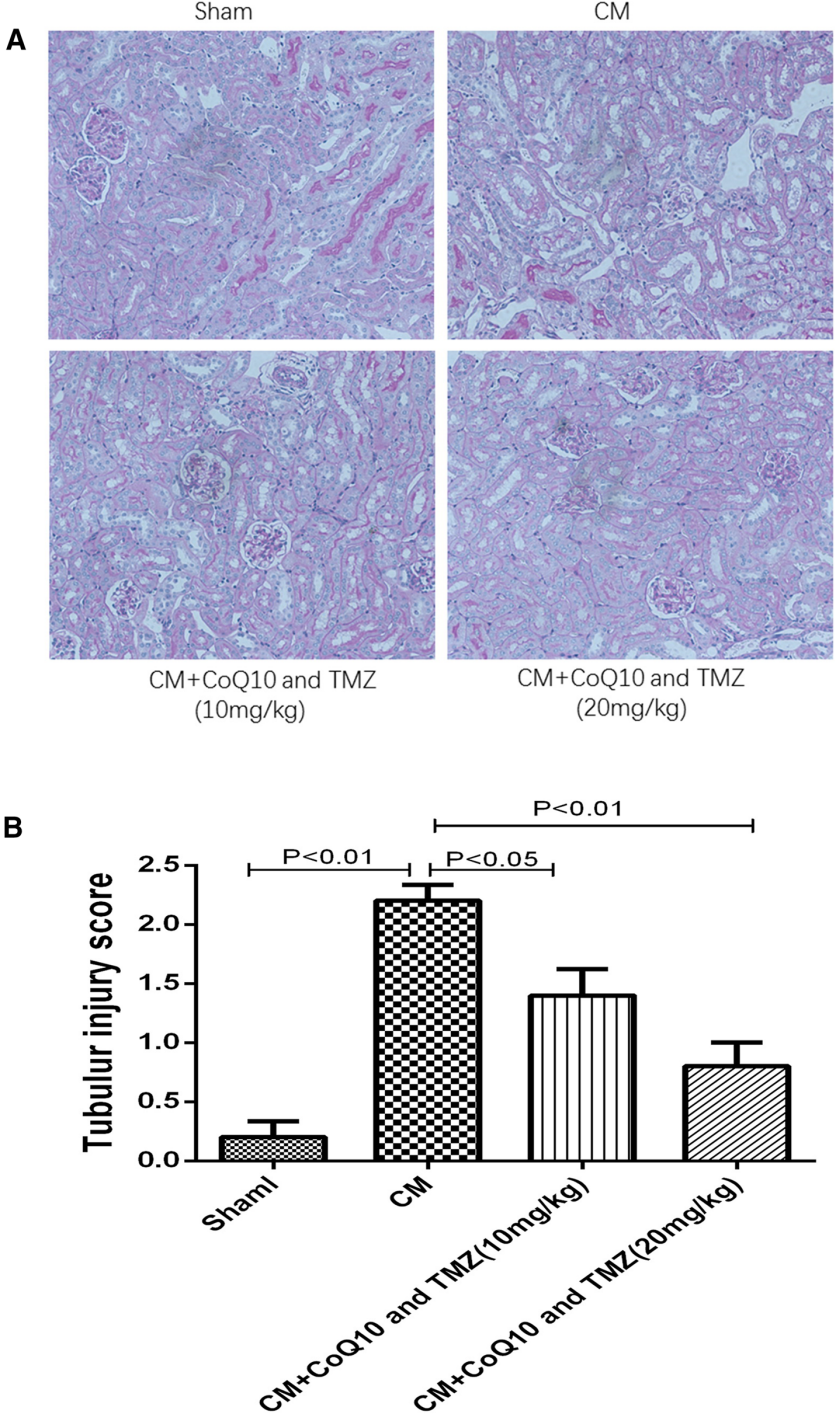
Effect of CoQ10 plus TMZ on contrast medium-affected renal tissues of rats by light microscopic examination (PAS). **a** Representative figures for each group. CM group had severe tubular damage with acute tubular necrosis, wide tubular epithelial vacuolation, and apoptotic tubular epithelium, while CoQ10 plus TMZ could reduce this pathological injuries. **b** Renal injury score. Each renal injury score value represents the mean of eight rats. All the pictures were taken under ×200 magnification

H&E staining for each kidney sections was performed to investigate the effect of CoQ10 and TMZ on cisplatin-induced renal tubular damage under light microscopy (×200 magnification). Serious tubular injuries were evident in the cisplatin-treated group. The main pathological characteristics included atrophy and degeneration of epithelial cells, hyaline materials and cast formation in the tubular lumen, and apoptosis and necrosis of tubular cells. After treatment with CoQ10 and TMZ, the damage was limited to mild necrosis of the tubular epithelial cells, slight epithelial swelling, and less cast formation was visible (Fig. 2a). Treatment with CoQ10 and TMZ significantly reduced the tubular necrosis score dose dependently compared to the score from CM-treated mice (Fig. 2b).

## Discussions

This study indicates that receiving CoQ10 plus TMZ treatment before procedure could reduce the incidence of CIN for the patients with renal insufficiency undergoing elective cardiac catheterization. In addition, we evaluated risk factors in these patients for CIN development and observed that the volume of contrast medium and decreased eGFR were independent correlates of CIN. Moreover, we found that administration of CoQ10 plus TMZ was an independent protective factor against CIN by multiple Logistic regression analysis. This may provide us with a new strategy for the prevention and treatment of CIN.

CIN is an iatrogenic renal injury that follows the contrast medium exposure, which developed in 48–72 h. It could cause transient increase in SCr [16, 17]. Currently, despite prevention strategies advances, CIN remains responsible for approximately 10% of all hospital-acquired acute kidney injury [18, 19]. Moreover, patients developing CIN are at a high risk of both a further deterioration of kidney function and an adverse clinical outcome following CAG or PCI [19].

At present, the pathophysiology of CIN is still not very clear—two possible mechanisms may be involved: (1) the direct cytotoxicity of contrast media (CM) to renal medulla, which injures vascular endothelial cells and renal tubular, leads to production of reactive oxygen species (ROS), reduces production of nitric oxide (NO), and finally causes vasoconstriction [20]; (2) The high viscosity and high permeability of CM results in slowing flow of renal medullary blood, which eventually leads to renal medulla ischemia [21]. Hypoxia, vasoconstriction, and cytotoxicity are the major adverse effects of contrast media. The direct toxicity of contrast media to renal tubular epithelial cells and the caused apoptosis have been confirmed by many clinical trials and animal experiments [22]. Reactive oxygen species (ROS) and oxidative stress play an important role in the apoptosis of renal tubular epithelial cells induced by contrast media.

Coenzyme Q10 (CoQ10) is a lipid-soluble benzoquinone and has 10 isoprenyl units in its side chain. It is a very important component of the mitochondrial respiratory chain for adenosine triphosphate synthesis. Besides, CoQ10 is also an intracellular antioxidant that protects mitochondrial membrane protein, the membrane phospholipids, and low-density lipoprotein cholesterol (LDL-c) from free radical-induced oxidative damage [23]. Literature has shown that CoQ10 protects cardiovascular by reducing oxidative stress and reducing low-density lipoprotein (LDL), thereby reducing the risk of coronary artery disease [24].

Trimetazidine hydrochloride (TMZ) is an anti-myocardial ischemia drug. It can improve the cardiac function and increase the exercise tolerance in patients with ischemic cardiomyopathy. Researches have shown that TMZ has cell protective effects on anti-apoptosis of ischemia, reperfusion of myocardial cell, and anti-oxidation; it also can restore mitochondrial membrane stability, reduce Ca^2+^ ion-induced mitochondrial damage, and restore glutathione peroxidase level [25, 26]. In addition, TMZ can improve the utilization rate of lactic acid, decrease ketogenesis in cells, improve the lipid metabolism, and inhibit the reduction of effective circulating blood volume and renal insufficiency caused by acidosis [27, 28].

Few studies focused on the preventive effects of CoQ10 and TMZ on CIN. As far as we know, this study might be the first one to demonstrate that the combination therapy of CoQ10 and TMZ can reduce the incidence of CIN in patients with renal insufficiency undergoing elective cardiac catheterization. Although Onbasili [29] and Shehata [30] have demonstrated that TMZ could reduce the acute kidney injury caused by nephrotoxic drugs and reduce the incidence of CIN, there is still controversy about the value of TMZ in the prevention of CIN. In the current study, we used Chinese population to perform a two-blind clinical trial, and confirm their efficiency.

With the aim to further confirm renal protective effect of CoQ10 and TMZ on CIN, in vivo animal experiment study was performed. Renal function test demonstrated that CoQ10 and TMZ could significantly reduce the BUN and SCR induced by CM injection, as well as attenuating tubular injury by pathological analysis. Oxidation assay suggests that CoQ10 and TMZ could significantly reduce the LPO, and increase the GSH and enzyme activity of SOD and CAT. All these results support our clinical trial results that CoQ10 and TMZ could ameliorate the CIN due to their anti-oxidation effect.

The exact mechanism of CoQ10 combined TMZ into prevention of CIN remains unclear. The most certain is that the combination of CoQ10 and TMZ has a strong anti-oxidation effect, and oxidative stress is one of the important pathogenesis of CIN. CoQ10 and TMZ attenuate oxidative stress and protect blood endothelial cells via activating AMPK and ERK pathways, or Akt/eNOS signaling pathways [25, 31, 32]. All these studies support that combination of CoQ10 and TMZ may have preventive effects on CIN.

There are some limitations and shortcomings in this study. First, it is a single-center study with small data samples, which may weaken the statistical power of the conclusions; Second, this study did not evaluate the preventive effect of CoQ10 and TMZ on CIN separately, and therefore further study is still needed in the future to explore the prevention of CoQ10 or TMZ alone on the CIN and their comparison to *N*-acetylcysteine, which has clear clinical curative effect.

## Conclusions

In conclusion, we demonstrated that oral CoQ10 plus TMZ could decrease the incidence of CIN in patients with renal insufficiency undergoing elective cardiac catheterization. The combination of CoQ10 and TMZ observed in this study suggests the therapeutic potential of the two drugs on prevention of CIN.
